# A sensitive mitochondrial thermometry 2.0 and the availability of thermogenic capacity of brown adipocyte

**DOI:** 10.3389/fphys.2022.977431

**Published:** 2022-08-24

**Authors:** Xiao-Yan Meng, Dian-Dian Wang, Tao-Rong Xie, Run-Zhou Yang, Chun-Feng Liu, Dan-Hua Liu, Shu-Ang Li, Yi Luan, Jian-Sheng Kang

**Affiliations:** ^1^ Clinical Systems Biology Laboratories, The First Affiliated Hospital of Zhengzhou University, Zhengzhou, China; ^2^ Department of Neurology, The First Affiliated Hospital of Zhengzhou University, Zhengzhou, China; ^3^ The Academy of Medical Sciences, Zhengzhou University, Zhengzhou, China; ^4^ Institute of Neuroscience, State Key Laboratory of Neuroscience, CAS Center for Excellence in Brain Science and Intelligence, Chinese Academy of Sciences, Shanghai, China; ^5^ Shanghai Center for Brain Science and Brain-Inspired Intelligence Technology, Shanghai, China; ^6^ RevoImmune Therapeutics Co Ltd., Shanghai, China

**Keywords:** thermogenesis, brown adipocytes, mitochondrial thermometry 2.0, TMRM, Rh800

## Abstract

The temperature of a living cell is a crucial parameter for cellular events, such as cell division, gene expressions, enzyme activities and metabolism. We previously developed a quantifiable mitochondrial thermometry 1.0 based on rhodamine B methyl ester (RhB-ME) and rhodamine 800 (Rh800), and the theory for mitochondrial thermogenesis. Given that the synthesized RhB-ME is not readily available, thus, a convenient mitochondrial thermometry 2.0 based on tetra-methyl rhodamine methyl ester (TMRM) and Rh800 for the thermogenic study of brown adipocyte was further evolved. The fluorescence of TMRM is more sensitive (∼1.4 times) to temperature than that of RhB-ME, then the TMRM-based mito-thermometry 2.0 was validated and used for the qualitatively dynamic profiles for mitochondrial thermogenic responses and mitochondrial membrane potential in living cells simultaneously. Furthermore, our results demonstrated that the heterogenous thermogenesis evoked by β3 adrenoceptor agonist only used overall up to ∼46% of the thermogenic capacity evoked by CCCP stimulation. On the other hand, the results demonstrated that the maximum thermogenesis evoked by NE and oligomycin A used up to ∼79% of the thermogenic capacity, which suggested the maximum thermogenic capacity under physiological conditions by inhibiting the proton-ATPase function of the mitochondrial complex V, such as under the cold activation of sympathetic nerve and the co-release of sympathetic transmitters.

## Introduction

Obesity is a global epidemic result from an imbalance between energy intake and expenditure (Blüher, 2019). The adipose-tissue pool in mammals is usually classified into three functionally different types: white, beige and brown, or further 8–10 subpopulations in human or mouse adipose tissues by single-nucleus RNA-sequencing (Sun et al., 2020). White adipose tissue is the primary site of energy storage (Barbosa et al., 2018). Brown adipose tissue and beige cells are important for both basal and inducible energy expenditure in the form of heat produced by the activation of uncoupling protein 1 (Ricquier, 2017). Because of its capacity for uncoupling mitochondria respiration, brown adipose tissue is an important site of energy expenditure (Jung et al., 2019). This led to the speculations that the function of brown adipocyte could be used to prevent obesity, diabetes, and related metabolic disorders (Lidell et al., 2014; Soler-Vázquez et al., 2018; Quan et al., 2020). To facilitate the research in this field, we previously developed a quantitative RhB-ME-based mitochondrial thermometry (mito-thermometry) 1.0 for simultaneously studying the thermogenic efficacy and mitochondrial membrane potential (MMP) of the brown adipocytes isolated from the interscapular brown adipose tissue of mice (Xie et al., 2017a; Xie et al., 2017b). Our previous works also demonstrated that self-restriction is a thermogenic characteristic of brown adipocyte, especially its thermogenic rate and capacity are limited by its proton pool or sources (Kang, 2018).

For convenience, considering that RhB-ME requires *de novo* synthesis (Xie et al., 2017a) and that TMRM is commercially available, we further evolved a mito-thermometry 2.0 based on TMRM and Rh800 for the thermogenic study of brown adipocyte. We demonstrated that the temperature sensitivity of RhB-ME was inferior to TMRM with three lines of evidence: thermal spectrum, ambient temperature, and endogenous thermogenic responses evoked by CCCP, CL316,243 or succinate.

## Materials and methods

### Animal ethics

All institutional and national guidelines for the care and use of laboratory animals were followed.

### The spectral characteristics of rhodamine dyes

The spectroscopic properties of rhodamine dyes were measured in an aqueous solution of 10 μM with fluorescence spectrometer (Varian Cary Eclipse). The excitation spectra of RhB, RhB-ME, TMRM were swept from 450 to 600 nm, and the emission wavelength were fixed at 620 nm. The emission spectra of RhB, RhB-ME and TMRM were swept from 550 to 700 nm, and the excitation wavelength were fixed at 530 nm. The excitation spectrum of Rh800 was swept from 500 to 740 nm, and the emission wavelength was fixed at 760 nm. The emission spectrum of Rh800 was swept from 650 to 850 nm, and the excitation wavelength was fixed at 630 nm. The excitation spectrums of Rh123 and Rh110 were swept from 400 to 540 nm, and the emission wavelength were fixed at 560 nm. The emission spectrums of Rh123 and Rh110 were swept from 490 to 650 nm, and the excitation wavelength were fixed at 470 nm. The pH sensitivity of rhodamine dyes was measured in solution pH ranging from 5.5 to 8.5 (all spectral data were normalized with peak values at pH 7.5), and the temperature sensitivity of rhodamine dyes was tested at different temperature ranged from 5 to 45°C (all spectral data were normalized with peak values at 25°C) with the same settings as the spectral analyses.

### Mitochondrial localization of rhodamine dyes

The evaluation of the mitochondrial localization of rhodamine dyes was studied in COS7 cells. Plasmid N1-Tag-mito-GFP or N1-Tag-mito-RFP was transfected into COS7 cells by calcium phosphate transfection method to mark mitochondria. According to the spectral characteristics of rhodamine dye, cells with mito-GFP expression were stained with RhB, RhB-ME, TMRM, TMRE or Rh800, and cells with mito-RFP expression were stained with Rh123 or Rh100. The final concentration of dyes was 50 nM. Mitochondrial localization imaging was performed with laser confocal microscope (ZEISS, LSM 980) with 40×/0.95 Plan-Apochromat objective. The pseudo-color of mito-GFP was green (excited at 488 nm and collected at 490–540 nm), the channel of RhB, RhB-ME, TMRM or TMRE was represented as red (excited at 549 nm and collected at 550–590 nm), and Rh800 channel was also shown in red (excited at 639 nm and collected at 640–735 nm). The pseudo-color of Rh123 and Rh110 was green (excited at 488 nm and collected at 490–540 nm), and the channel of mito-RFP was represented as red (excited at 549 nm and collected at 550–590 nm). The pinhole of all channels was size 1, and the size of 16-bit images was 2048 × 2048 pixels.

### The isolation and culture of primary brown adipocyte

Brown adipocytes were isolated from C57BL/6J male mice aged 18–24 days, all mice were purchased from Charles River, China. The method of brown adipocytes separation was optimized on the basis of procedure by Xie et al. (2017b). The 18-day-old mice were exposed at 4°C for 10 h. For mice over 18 days, the duration of cold treatment increased by an hour for each additional day. After mice were sacrificed by cervical dislocation, interscapular adipocyte tissue (BAT) was quickly dissected and placed in pre-cooled phosphate buffered saline. White adipose tissue, fascia, blood vessels and nerves were removed under a stereoscopic microscope (Olympus, MVX10). The BAT was rinsed for 3 times in the bio-safety cabinet with 5 ml dissociation solution (DS: 4% newborn calf serum in Dulbecco’s modified eagle medium, abbreviated as DMEM) and cut into ∼ 1 mm size. The BAT fragments were digested by 0.2% collagenase II in a shaking water bath at 37°C for 30 min. Let the mixture settle for 2 min. Discard supernatant and resuspend pellets in 5 ml DS, standing for another 2 min. Precipitation was resuspended by pipetting up and down 10 times with 2 ml DS using the 1 ml pipette (Eppendorf, #0030073.100). The diameter (∼850 μm) of the 1 ml Eppendorf pipette is relatively wide and sufficient to effectively avoid large tissue blocks and shear BAT into small remains. Add 3 ml DS to the suspension and settle for 5 min, then collect the supernatant into a new centrifuge tube. Resuspend the precipitated pellets and pipette 10 times in 1 ml of DS using the 1 ml pipette (Axygen, #T-1000-B), and add DS to 3 ml and let it settle for 5 min. The diameter (∼700 μm) of the 1 ml Axygen pipette is relatively narrow and effective for breaking the remaining small tissues into individual-isolated brown adipocytes. The pipettes with narrower diameters were avoided since they might easily break the cells. Added DS to 3 ml and let it stand for 5 min. The brown adipocytes supernatant was transferred into the same tube and centrifuged for 800 g for 5 min at 4°C. The centrifuged brown adipocytes washed with PBS, centrifuged at 650 g for 5 min at 4°C, then re-suspended in the plating medium (PM: 5% fetal bovine serum/DMEM supplemented with 4 μM cytosine arabinoside (Ara-C, Sigma, C6645) to inhibit fibroblast proliferation) with 200 μL PM per mouse, and aliquot the cell suspension into 100 microliters per coverslip (12 mm size, No. 0 thickness, Carolina Biological Supply P-763–3,009) pre-coated with matrigel in 35 mm dishes. Finally, add 2 ml PM to the dishes after 3–4 h. Cells were cultured at 37°C in a humidified constant temperature incubator with 95% air and 5% CO_2_. Exchange half medium every 2 days and gently take away the lipid droplets floating on the surface of the brown adipocytes. Imaging experiments were usually performed with the primary brown adipocytes cultured for 4–7 days.

### Rhodamine dyes under different environmental temperatures

The fluorescent imaging was performed using a laser confocal microscope (ZEISS, LSM 980) with 40×/0.95 Plan-Apochromat objective for representative images at 27 and 37°C. brown adipocytes were co-stained with Rh800 and TMRM or RhB-ME (all dyes used at the concentration of 50 nM) in Tyrode’s solution (amount for 1 L in gram: 6.953 NaCl, 0.372 KCl, 4.775 HEPES, 5.4 Glucose, 9.75 Sucrose, 0.184 MgCl2, 0.222 CaCl2, adjust pH to 7.4 with 10 mM NaOH, Filtered) at 37°C for at least 30 min. Zeiss imaging incubator was used to control temperature at 27°C or 37°C, the definite focus of LSM 980 was used to correct for the focal drift caused by the temperature change. The pseudo-color of Rh800 channel was red (excited at 639 nm and collected at 640–735 nm), and the channel of TMRM or RhB-ME represented as green (excited at 549 nm and collected at 550–590 nm). The pinholes of all channels were set to maximum, and the size of 16-bit images was 1,024 × 1,024 pixels.

### Endogenous thermogenesis

Time-lapse imagings and representative images were performed using a laser confocal microscope (ZEISS, LSM 980) with 20×/0.8 Plan-Apochromat objective or ×40/0.95 Plan-Apochromat objective, respectively. Cells were co-stained Rh800 with TMRM or RhB-ME (all dyes used at the concentration of 20 nM) in Tyrode’s solution at 37°C for at least 30 min. The pseudo-color of Rh800 channel was red (excited at 633 nm and collected at 635–735 nm), and the channel of TMRM or RhB-ME was green (excited at 555 nm and collected at 560–601 nm). The pinholes of all channels were set to maximum, the size of 12-bit images was 512 × 512 pixels. 120 frames were recorded at an interval of 20 s in time series imaging. CCCP (5 μM), NE (0.1 μM), CL316,243 (0.1 μM) and succinate (10 mM) were injected into the Tyrode’s solution at the 16th frame during imaging. Oligomycin (10 μg ml^−1^) was used to pretreat brown adipocytes for 5 min before the time-lapse imaging.

### Theoretical derivation for the temperature sensitivity of the TMRM-based or RhB-ME-based mito-thermometry

According to Arrhenius equation, the fluorescent intensity ***I*** of mitochondrial-targeting rhodamine dyes at temperature ***T*** can be fitted with:

$$I=Ae^{-\frac{E_{a}}{RT}}$$ (1)

where ***R*** is the gas constant; ***E***_***a***_ is the activation energy of the rhodamine dye, which can be experimentally estimated; ***T*** is the kelvin temperature; ***A*** is a parameter related to imaging setup and experimental settings.

The parameter ***A*** in Equation 1 can be canceled out by dividing the fluorescent intensity ***I***_***ref***_ of a reference region outside of the cell with a measurable temperature ***T***_***ref***_. Thus, the normalized ratio ***r*** is a function of ***T*** and determined by Equation 2:

$$r=\frac{I}{I_{ref}}=e^{-\frac{E_{a}}{R}\left(\frac{1}{T}-\frac{1}{T_{ref}}\right)}$$ (2)

Considering Rh800 is insensitive to temperature, the activation energy of Rh800 is zero, so that the thermal ratios of Rh800 to TMRM or RhB-ME are written as follows:

$$\frac{r_{Rh800}}{r_{TMRM}}=e^{\frac{E_{a\_TMRM}}{R}\left(\frac{1}{T}-\frac{1}{T_{ref}}\right)}$$ (3)

$$\frac{r_{Rh800}}{r_{RhB-ME}}=e^{\frac{E_{a\_RhB-ME}}{R}\left(\frac{1}{T}-\frac{1}{T_{ref}}\right)}$$ (4)

Mitochondria with large negative membrane potentials lead to spontaneous accumulation of thermosensitive RhB-ME or TMRM and thermoneutral Rh800 in their matrixes, until reaching equilibrium in accordance with the Nernst distribution law:

$$\psi =-\frac{RT}{F}\ln\left(\frac{I_{mito}}{I_{ref}}\right)=-\frac{RT}{F}\ln\left(r\right)$$ (5)

where ***F*** is the Faraday constant; ψ is the mitochondrial membrane potential (MMP).

Since Rh800 is insensitive to the change of temperature, the ψ value of Rh800 can be taken as a true ψ value of MMP. The ψ value difference between Rh800 and TMRM or RhB-ME contains the information of temperature since their fluorescent intensity ***I*** and ratios to Rh800 are sensitive to temperature. The deviation of MMP estimation using TMRM or RhB-ME can be written as follows:

$$\Delta \psi_{TMRM}=\psi_{Rh800}-\psi_{TMRM}=-\frac{RT}{F}\ln\left(\frac{r_{Rh800}}{r_{TMRM}}\right)$$ (6)

$$\Delta \psi_{RhB-ME}=\psi_{Rh800}-\psi_{RhB-ME}=-\frac{RT}{F}\ln\left(\frac{r_{Rh800}}{r_{RhB-ME}}\right)$$ (7)

Equation 3 and Equation 4, Equation 6, and Equation 7 yield the following:

$$\Delta T_{TMRM}=T_{ref}\frac{F}{E_{a\_TMRM}}\Delta \psi_{TMRM}$$ (8)

$$\Delta T_{RhB-ME}=T_{ref}\frac{F}{E_{a_{\_RhB-ME}}}\Delta \psi_{RhB-ME}$$ (9)

Equation 8 and Equation 9 show that the deviation of MMP estimation using thermosensitive TMRM or RhB-ME is linearly proportional to the change or difference of temperature, which suggests that TMRM and RhB-ME should not be used for measuring MMP. In addition, Equation 8 and Equation 9 also indicate that the temperature sensitivity of TMRM or RhB-ME is inversely related to their activation energy (-3.2 kcal/mol and -4.4 kcal/mol respectively), which is consistent with the thermochromic mechanism of RhB-ME (Xie et al., 2017a) or TMRM (Figure 2).

## Results

### The characteristics of small rhodamine dyes

**FIGURE 1:**
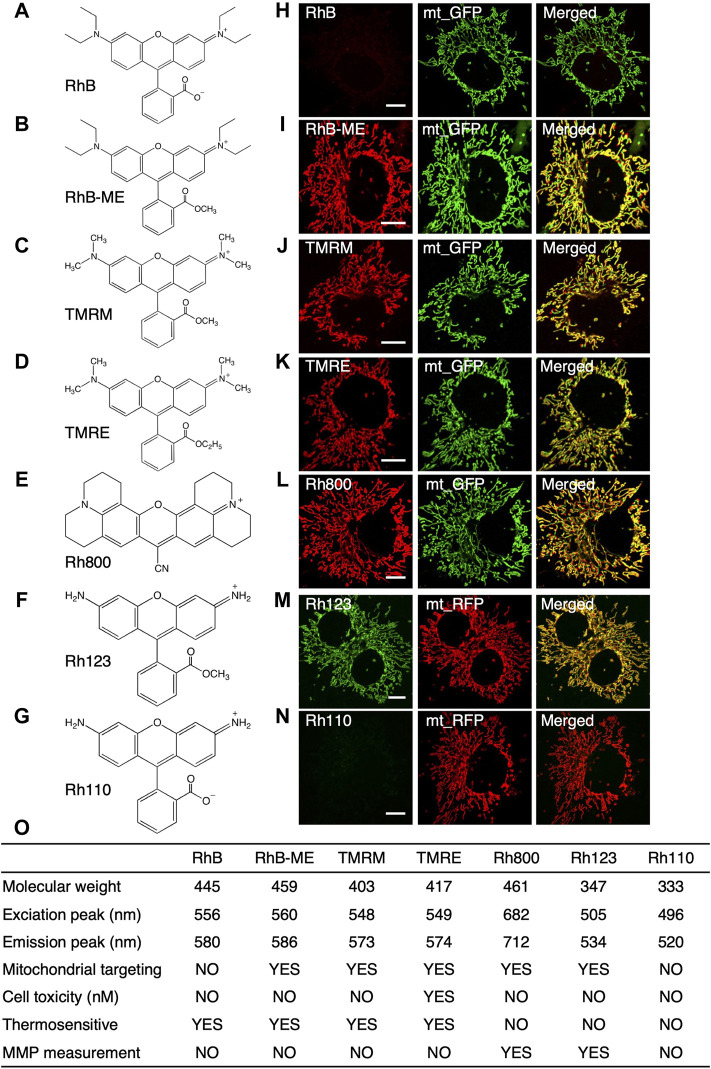
The summary for the characteristics of rhodamine dyes. **(A–G)**, the structure of rhodamine dyes. **(A)**, RhB (rhodamine B). **(B)**, RhB-ME (rhodamine B methyl ester). **(C)**, TMRM (tetra-methyl rhodamine methyl ester). **(D)**, TMRE (tetra-methyl rhodamine ethyl ester). **(E)**, Rh800 (rhodamine 800). **(F)**, Rh123 (rhodamine 123). **(G)**, Rh110 (rhodamine 110). **(H–N)**, Co-localization of rhodamine dyes with mt-GFP or mt-RFP in COS7 cells. RhB **(H)** and Rh110 **(N)** are unable to target in mitochondria, while RhB-ME **(I)**, TMRM **(J)**, TMRE **(K)**, Rh800 **(L)** and Rh123 **(M)** target mitochondria very well. **(O)**, the summary of the characteristics of rhodamine dyes.

For a more sensitive mito-thermometry 2.0, we systematically explored the temperature sensitive characteristics of rhodamine dyes (Figure 1) since that rhodamines had excellent properties such as high fluorescence quantum yield, low cost, low toxicity, excellent rigid planar structure and their stable spectrum in the visible light (Zheng et al., 2013). For the convenience of experiments, only those frequently-used and commercially available rhodamine dyes with small molecule weights (<500 Da) were screened, summarized and discussed (Figure 1), since large rhodamine dyes were usually slow in cell permeability and transmembrane diffusion (Matsson and Kihlberg, 2017), such as Mito-RTP (Homma et al., 2015). Large rhodamine dyes might also have unknown effects or artifacts, such as Mito thermo yellow, which could unfavorably bind to aldehyde dehydrogenase (Kim et al., 2011; Chrétien et al., 2020).

**FIGURE 2:**
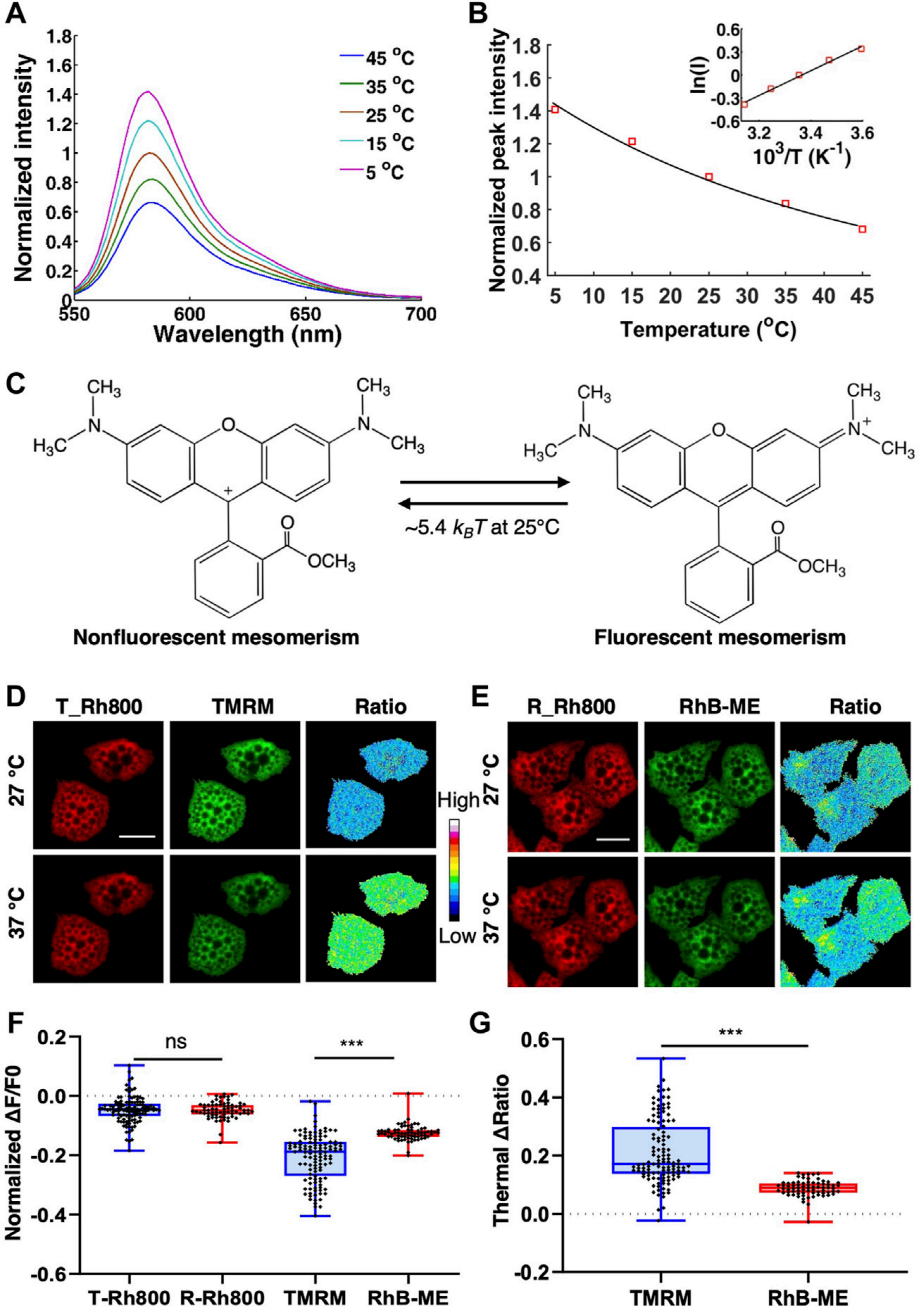
The thermochromic mechanism and temperature sensitivity of TMRM. **(A)**, the emission spectra of 10 μM TMRM (solid lines) from 5 to 45°C, respectively. **(B)**, the Arrhenius plot for TMRM thermochromic transformation in aqueous solution. The black solid line indicates the Arrhenius fitting to the peak values (red square) of TMRM data in panel A. The inset shows the Arrhenius plot. **(C)**, the thermal energy (∼5.4 kBT at 25°C) converts single TMRM fluorescent molecule to its nonfluorescent form. D-G, brown adipocytes were co-stained with 50 nM of Rh800 and 50 nM of TMRM **(D)**, or 50 nM of Rh800 and 50 nM of RhB-ME **(E)**, and then incubated at 27°C or 37°C. **(D,E)**, the representative fluorescence images and thermal ratio images of brown adipocytes at 27°C or 37°C. The pseudo-color ratio of Rh800 to TMRM **(D)** or Rh800 to RhB-ME **(E)** represented thermal status. Scale bars, 20 μm. **(F)**, the scatter plot with boxplots showed the changes in fluorescent intensity of RH800, TMRM and RhB-ME from 27 to 37°C. The results showed that the fluorescent intensity of Rh800 co-stained with TMRM (T_Rh800, n = 120) and co-stained with RhB-ME (R_Rh800, n = 71) changed slightly after increasing temperature. The fluorescence intensity of TMRM (n = 120) dropped significantly more than that of RhB-ME (n = 71). **(G)**, the scatter plot with boxplots showed the thermal ratio of Rh800 to TMRM (n = 120) was significantly larger than the ratio of Rh800 to RhB-ME (n = 71). Box and whiskers. Min to Max. Each point represented a single cell.

As summarized in the table (Figure 1O), the spectral properties of rhodamine dyes (Supplementary Datas S1–S3) and the fluorescence intensity of rhodamine dyes were not affected by pH (Supplementary Data S2); the fluorescence intensity of rhodamine B (RhB, Figure 1A, Supplementary Data S3A), RhB-ME (Figure 1B, Supplementary Data S3B), TMRM (Figure 1C, Figure 2A) and tetra-methyl rhodamine ethyl ester (TMRE, Figure 1D) were decreased as temperature increased, and rhodamine 800 (Rh800, Figure 1E, Supplementary Data S3C), rhodamine 123 (Rh123, Figure 1F) and rhodamine 110 (Rh110, Figure 1G, Supplementary Data S3D) were temperature insensitive. RhB (Figure 1A) and Rh110 (Figure 1G) existed as zwitterions at physiologic pH, which were cell membrane impermeable (Johnson et al., 1981; Xie et al., 2017a), and we also experimentally demonstrated (Figures 1H–N) that they had no capability for mitochondrial targeting. TMRE at nano-molar could inhibit mitochondrial function (Scaduto and Grotyohann, 1999; Liu et al., 2016), so that we previously utilized TMRE to model neural atrophy and used TMRM as a nontoxic experimental control (Liu et al., 2016). Rh800 and Rh123 were insensitive to temperature and well suited for representing mitochondrial membrane potential (MMP) because their accumulation in mitochondrial matrix according to the Nernst distribution law. Although TMRM was commonly used for measuring MMP(Chazotte, 2011), The deviation of MMP estimation using RhB-ME or TMRM was linearly proportional to the change or difference of temperature (see the Equation 8 and (9) in the *Theoretical derivation for the temperature sensitivity of the TMRM-based or RhB-ME-based mito-thermometry* Section). Therefore, the thermosensitive RhB-ME (Supplementary Data S3B) and TMRM (Figures 1, 2) were not appropriate to quantify MMP (see the conclusion of the *Theoretical derivation for the temperature sensitivity of the TMRM-based or RhB-ME-based mito-thermometry* Section).

### A supersensitive TMRM-based mito-thermometry 2.0

RhB-ME and TMRM are membrane permeable, and spontaneously accumulated in mitochondria, both of which equilibrated as a thermosensitive mixture of a nonfluorescent form and a fluorescent resonance form. Thermal energy (∼7.5 k_B_T at 25°C) can convert single RhB-ME fluorescent form to its nonfluorescent form (Xie et al., 2017a). The thermochromic activation energy of RhB-ME was contributed to the torsional motion of diethylamino groups (Xie et al., 2017a). Because that the dimethylamino groups of TMRM are smaller than the diethylamino groups of RhB-ME, TMRM is more sensitive to the temperature change (Figure 2). Indeed, Arrhenius plot (Figure 2B) indicated that the activation energy of TMRM thermochromic transformation (Figure 2A) was about -3.2 kcal/mol, which was less than the activation energy of RhB-ME (-4.4 kcal/mol) (Xie et al., 2017a). Consequently, a lower thermal energy (∼5.4 k_B_T at 25°C) could convert single TMRM fluorescent form to its nonfluorescent form (Figure 2C). For qualitative thermogenic studies, TMRM was recommended since it was commercially available and more thermosensitive than RhB-ME.

To compare the sensitivity of TMRM and RhB-ME in living cells, we first used warming or cooling experiments to check the fluorescent changes of TMRM and RhB-ME in the primary cultured brown adipocytes co-stained Rh800 with TMRM or RhB-ME (Figures 2D–G). Being consistent with the thermal insensitivity of Rh800 (Figure 1H), the fluorescent intensities of Rh800 in TMRM and RhB-ME stained group showed no difference as expected (T-Rh800, -0.05 ± 0.05 and R-Rh800, -0.05 ± 0.03, in mean ± standard deviation (SD), respectively, Figures 2D–F). The small decreased fluorescence of Rh800 in higher temperature (37°C) might just reflect a slightly lower MMP at 37°C due to an increased mitochondrial metabolism at 37 versus 27°C (Figures 2D–F). Meanwhile, the fluorescence (in mean ± SD) of TMRM (-0.21 ± 0.08) or RhB-ME (-0.12 ± 0.03) was clearly dropped, and the normalized fluorescent change of TMRM was decreased significantly (*t tests, p* = 2.87 × 10^−20^) more than that of RhB-ME at 37°C (Figures 2D–F). Therefore, the ratio (in mean ± SD) of Rh800 fluorescent intensity (-0.21 ± 0.11) to TMRM (-0.09 ± 0.03) was significantly larger (*t tests, p* = 4.11 × 10^−22^) than the ratio of Rh800 to RhB-ME (Figure 2G). The ratios of Rh800 to TMRM or RhB-ME were used to represent as the mitochondrial thermal profile to cancel out the influence of MMP on the concentration and fluorescence of TMRM or RhB-ME (Xie et al., 2017a; Xie et al., 2017b; Kang, 2018).

### The validation of the TMRM-based mito-thermometry 2.0 in endogenous thermogenesis studying

**FIGURE 3:**
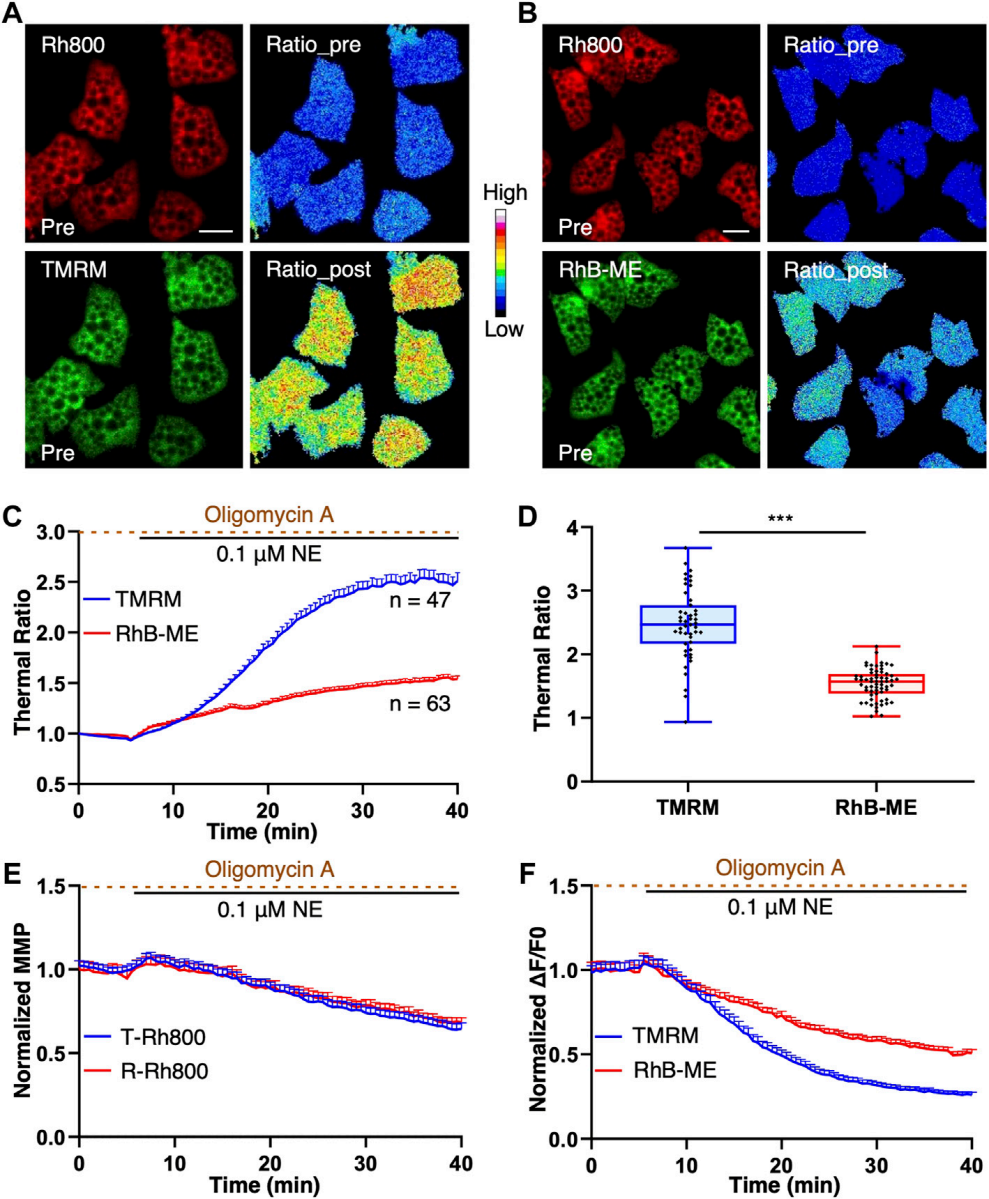
TMRM is more temperature thermosensitive than RhB-ME. **(A–F)**, brown adipocytes were co-stained with 20 nM of Rh800 and 20 nM of TMRM, or 20 nM Rh800 and 20 nM RhB-ME, and co-treated with 0.1 μM NE and 10 μg ml-1 oligomycin A after 5 min of imaging. **(A,B)**, the representative fluorescent images of brown adipocytes before the stimulation, and thermal ratio images before and after co-treatment. The pseudo-color ratios of Rh800 to TMRM **(A)** or Rh800 to RhB-ME **(B)** represented thermogenic responses. Scale bars, 20 μm. **(C)**, the thermal ratio of Rh800 and TMRM compared with the ratio of Rh800 and RhB-ME in brown adipocyte. After the co-treatments of NE and oligomycin A in brown adipocytes, the thermal ratios of Rh800 to TMRM (blue line, n = 47) changed more dramatical than the ratios of Rh800 to RhB-ME (red line, n = 63). **(D)**, the scatter plot with boxplots showed that the ratios of Rh800 to TMRM (blue box, n = 47) were generally greater than the ratios of Rh800 to RhB-ME (red box, n = 63) (t tests, *p* = 1.58 × 10^−15^). Box and whiskers. Min to Max. Each point represents a single cell. **(E)**, the fluorescent intensity of Rh800 represented the MMP dynamics of brown adipocytes. The results showed that the brown adipocytes co-stained Rh800 with TMRM (blue line, n = 47) or RhB-ME (red line, n = 63) had similar MMP dynamics. **(F)**, the comparison of the fluorescent intensity changes of TMRM (blue line, n = 47) or RhB-ME (red line, n = 63) in brown adipocytes. All data points represented mean +s.e.m.

To further verify and validate the thermal sensitivity of TMRM and RhB-ME (Figure 3) in the experiments of endogenous thermogenesis, both the TMRM-based and RhB-ME-based ratiometric mito-thermometries were further tested in the brown adipocytes cotreated with NE and oligomycin A. Oligomycin A can inhibit the functions of mitochondrial complex V, especially its ATPase and proton-pumping function invoked by NE, and thus enhance thermogenic response (Xie et al., 2017b). NE stimulation could activate the proton-ATPase function of the mitochondrial complex V, which led to the phenomenon that brown adipocytes had two MMP subpopulations (depolarization and hyperpolarization) and formed the futile cycle with uncoupling protein 1 to inhibit thermogenesis (Xie et al., 2017a; Xie et al., 2017b). Consequently, only about half of brown adipocytes stimulated by NE showed thermogenic responses (Xie et al., 2017a; Xie et al., 2017b) (Supplementary Data S4), while almost all of brown adipocytes treated with NE + oligomycin A showed thermogenic responses (Xie et al., 2017b). Thus, the NE + oligomycin A combination rather than NE alone was used as the stimulant to induce thermogenesis in brown adipocytes to avoid the potential influences of heterogeneously thermogenic responses.

The basal distribution and fluorescent intensity of TMRM and RhB-ME in brown adipocytes were comparable and stable (Figures 3A,B). After the NE + oligomycin A stimulation, the fluorescent intensity of brown adipocytes stained with TMRM decreased significantly more than that of RhB-ME (Figure 3C), meanwhile the MMP change represented by the fluorescent intensity of Rh800 in the brown adipocytes stained with TMRM was similar to the intensity of Rh800 stained with RhB-ME (T-Rh800 and R-Rh800 respectively, Figure 3E). The thermal ratio values of the brown adipocytes stained with TMRM (2.51 ± 0.55) increased ∼1.6 times (163%) than the values of RhB-ME (1.54 ± 0.24) (Figures 3D,F) under the same stimulation, indicating that TMRM was much more thermosensitive. Theoretically, the thermal sensitivity of TMRM was ∼1.4 times than that of RhB-ME according to their ratio of the activation energies (-3.2 kcal/mol and -4.4 kcal/mol respectively) (the theoretical derivation in methods). The discrepancy of the thermal sensitivity between the experimental result and the theoretical calculation suggested that the extra ∼23% of sensitivity might be due to the lighter molecule weight (Dalton, Da) and smaller size (Figure 1H) of TMRM (403 Da) than that of Rh800 (461 Da, ∼15% heavier than TMRM), and thus its faster transmembrane diffusion under the depolarization (Figure 3D) of MMP after the stimulation. Meanwhile, the molecule weight or size of RhB-ME (459 Da) was comparable to that of Rh800 and only differed by 0.4% in molecule weight (Figure 1H). Thus, the RhB-ME-based mito-thermometry 1.0 was more appropriate and accurate for quantitative studies and theoretical deductions as demonstrated in our previous studies (Xie et al., 2017a; Xie et al., 2017b; Kang, 2018), while the TMRM-based mito-thermometry 2.0 was much more sensitive and convenient for qualitative experiments (Figures 3E,F).

### Applications of the mito-thermometry 2.0 in measuring the availability of thermogenic capacity of brown adipocyte under stimulations

**FIGURE 4:**
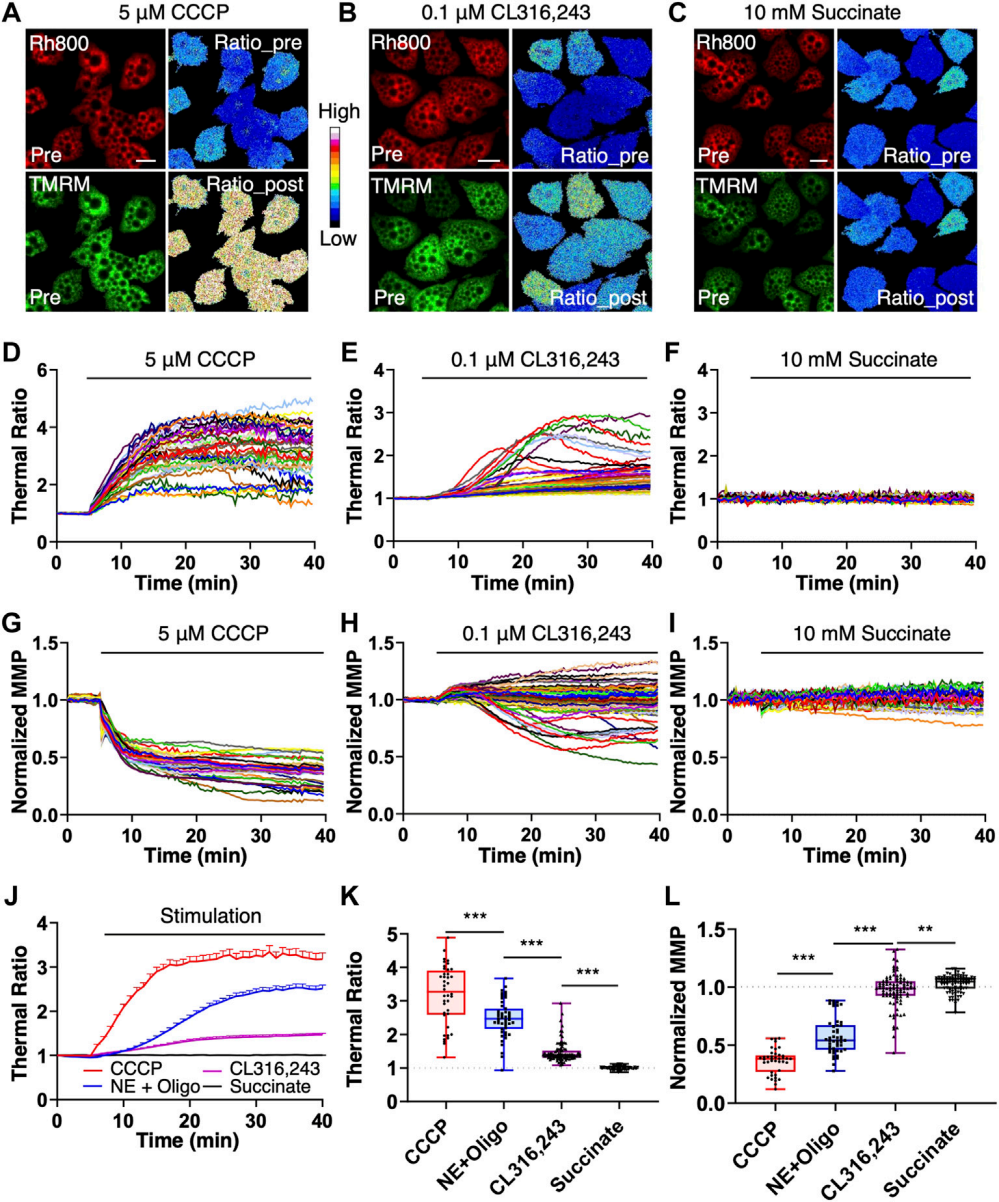
Endogenous thermogenic studies with the mitochondrial thermometry 2.0 in BA. **(A–F)**, the endogenous thermogenesis tests with the mitochondrial thermometry 2.0 in brown adipocytes. The pseudo-color ratios of Rh800 to TMRM represented thermogenic responses, and the fluorescent intensity of Rh800 represented MMP. After 5 min of live cell imaging for the base line under resting condition, brown adipocyte was treated with 5 μM of CCCP, 0.1 μM of NE with 10 μg ml-1 of oligomycin A pretreatment, 0.1 μM of CL316,243 or 10 mM of succinate. Mitochondrial thermogenic responses and MMP dynamics were simultaneously imaged for data analyses **(D–K)**. **(A–C)**, were representative fluorescent images (Rh800 and TMRM) and their pseudo-color thermal ratios of Rh800 to TMRM in brown adipocytes before and after the treatments of CCCP **(A)**, CL316,243 **(B)** or succinate **(C)**, respectively. Scale bars, 20 μm. **(D–F)**, the thermogenic responses in brown adipocytes evoked by CCCP [**(D)**, n = 41], CL316,243 [**(E)**, n = 91] or succinate [**(F)**, n = 87], respectively. Each colored trace represented a single brown adipocyte [in **(D–F)**]. **(A,D)**, the brown adipocytes treated with CCCP (n = 41) showed high thermogenic efficiency and large responses. **(B,E)**, demonstrated the relatively low thermogenic efficacy of CL316,243 (n = 91) in brown adipocytes. **(C,F)**, showed that succinate (n = 87) stimulation did not have thermogenic effect in brown adipocytes. **(G–I)**, the raw data plots of MMP changes in brown adipocytes treated with CCCP [**(H)**, n = 41], CL316,243 [**(I)**, n = 91] or succinate [**(G)**, n = 87]. Each colored curve represented a single brown adipocyte. **(G)**, the results showed the depolarization of MMP in brown adipocytes treated with CCCP (n = 41). **(H)**, there were two populations of MMP changes (hyperpolarization and depolarization) in brown adipocytes treated with CL316,243 (n = 91). **(I)**, succinate treatment didn’t affect MMP in brown adipocytes. **(J)**, the summary and comparison of the averaged thermogenic responses of brown adipocytes evoked by CCCP (red line, n = 41), NE and oligomycin A co-treatment (blue line, n = 47), CL316,243 (purple line, n = 91) or 10 mM succinate (black line, n = 87). **(K)**, scatter plotting the thermogenic ratio values with boxplots for CCCP (red box, n = 41), NE + Oligomycin A (blue box, n = 47), CL316,243 (purple box, n = 91) and succinate (black box, n = 87) treatments on brown adipocytes. **(L)**, scatter plotting the normalized MMP with boxplots under the stimulations of CCCP (red box, n = 41), NE + Oligomycin A (blue box, n = 47), CL316,243 (purple box, n = 91) and succinate (black box, n = 87). In **(K,L)**, each point represented a single brown adipocyte.

For further application of the mito-thermometry 2.0, we checked the thermogenic responses and MMP dynamics induced by an uncoupling reagent CCCP, a specific β3 adrenoceptor agonist CL316, 243 and succinate, a substrate in the tricarboxylic acid cycle (Figure 4). The results demonstrated that the mito-thermometry 2.0 was very sensitive to detect mitochondrial thermogenic responses in brown adipocytes (Figure 4). Compared to the homogeneous thermogenesis induced by CCCP and NE + Oligomycin A (Figures 4A,D, Figures 4J,K) and the heterogenous thermogenic responses under CL316,243 stimulation (Figures 4B,E, Figures 4J,K), succinate treatment in brown adipocytes did not evoke any thermogenic response (Figures 4C,F, Figures 4J,K).

In addition, contrasted with the homogeneous depolarization of MMP caused by CCCP (Figures 4G,L) and the two MMP subpopulations (hyperpolarization and depolarization) under CL316,243 stimulation (Figures 4H,L), succinate treatment in brown adipocytes almost did not change MMP (Figures 4I,L). Considering the supersensitivity of the mito-thermometry 2.0 and the above validations, the flattened responses of the brown adipocytes under 5 mM (data not shown) or 10 mM succinate treatment (Figures 4C,F and Figures 4I–L) were quite astonishing, which didn’t support the conclusion that succinate could protect against diet-induced obesity by activating the thermogenic effect of brown adipose tissue claimed by Mills et al.(Mills et al., 2018).

As demonstrated in Figure 4K, the results showed that CCCP had the strongest thermogenesis effect on brown adipocytes, and the thermogenesis efficiency of NE + Oligomycin A was slightly weaker (*t tests, p* = 1.89 × 10^−5^) than that of CCCP, but much stronger (*t tests, p* = 7.25 × 10^−17^) than that of CL316,243. However, succinate had no thermogenic effect (*t tests, p* = 7.75 × 10^−25^) on brown adipocytes, even compared to CL316,243 which has a weak heat-producing effect. In Figure 4L, CCCP induced a homogenous and the largest amplitude change of mitochondrial depolarization in brown adipocytes. NE + Oligomycin A also evoked strong depolarization, which was greater (*t tests, p* = 1.50 × 10^−29^) than that of CL316,243, but weaker (*t tests, p* = 1.46 × 10^−10^) than that of CCCP. Succinate also did not cause mitochondrial depolarization in brown adipocytes.

The amplitudes of thermogenic responses induced by CCCP or NE + oligomycin A (Figures 4J,K) are 3.18 ± 0.89 and 2.51 ± 0.55 in mean ± SD, respectively. The MMP under the stimulations of CCCP or NE + oligomycin A (Fig. 4L) depolarized to 36 ± 11% and 62 ± 15% (Supplementary Data S4) in mean ± SD, respectively. The results suggested that the maximum thermogenesis evoked by NE + oligomycin A could use up to ∼79% of the thermogenic capacity evoked by CCCP stimulation, while the MMP changes induced by NE + oligomycin A is ∼58% of the MMP depolarization induced by CCCP (Supplementary Data S4).

The amplitudes of thermogenic responses induced by CL316,243 or succinate (Figures 4J,K) are 1.47 ± 0.31 and 1.0 ± 0.05 in mean ± SD, respectively. The MMP under the stimulations of CL316,243 or succinate (Figure 4L) changed to 0.97 ± 0.15 and 1.03 ± 0.07 in mean ± SD, respectively. The results suggested that the thermogenesis evoked by the β3 adrenoceptor agonist CL316, 243 could only use up to ∼46% of the thermogenic capacity (evoked with CCCP stimulation), while the MMP depolarization induced by CL316, 243 is heterogenous (Figures 4H,L), and there was almost no MMP changes under succinate stimulation (Figures 4I,L).

## Discussion

Ratiometric measurements could avoid variations and influences including illumination intensity and membrane potentials. In addition, for thermal ratiometric measurements using two dyes, there were extra factors to be considered, such as colocalization, hydrophobicity or hydrophility, transmembrane diffusion and crosstalk between spectrums. Therefore, for monitoring mitochondrial thermogenesis, Rh800 rather than Rh123 was used as a reference dye since the spectrum of Rh800 could be well separated from others (Figure 1O). In our previous (Xie et al., 2017a; Xie et al., 2017b; Kang, 2018) and present studies, we demonstrated that the ratiometric mito-thermometry based on Rh800 with RhB-ME or TMRM could simultaneously detect MMP and mitochondrial temperature quantitatively or qualitatively respectively.

At present, many methods were used to monitor metabolic activities and then indirectly check thermogenesis, such as oxygen consumption rate (Mills et al., 2018; Tao et al., 2020), reactive oxygen species (Chouchani et al., 2016; Mills et al., 2018), redox (He et al., 2020), whereas the methods of mito-thermometry were seldom adopted for such purposes. The RhB-ME-based mito-thermometry 1.0 applied in our previous study about the metabolism of primary cultured hepatocyte was far more sensitive than other assays since it could detect the responses evoked by 10 nM Prostaglandin E2 while other assays need ∼1,000 times higher concentration of Prostaglandin E2 (Shen et al., 2018). Consequently, we were able to identify that prostaglandin E2 receptor 4 mediated the effects of Prostaglandin E2 in hepatocytes, such as the lipid accumulation (Shen et al., 2018).

In our present works, we validated that the temperature sensitivity of TMRM was superior to RhB-ME using three lines of evidence including activation energy (Figures 2A–C), ambient temperature (Figures 2D–G), and endogenous thermogenesis (Figure 3). As for endogenous thermogenesis induced by NE + Oligomycin A, TMRM-based mito-thermometry 2.0 showed a greater amplitude than that of the mito-thermometry 1.0, while their MMPs were similar (Figure 3). The supersensitive mito-thermometry 2.0 have made metabolic study quite convenient and facilitated qualitative assays, especially for brown and beige adipocytes. One of the limitations of the TMRM-based mito-thermometry 2.0 is that the fluorescence of these rhodamine dyes diminished after the large depolarization of MMP, which might cause difficulties in data analysis. For physiological stimulations, the changes of MMP are usually limited (about a half of MMP depolarization evoked by CCCP) (Figure 3, Figure 4 and Supplementary Data S4) so that the thermogenic results are solid enough especially for the quantizable RhB-ME-based mito-thermometry 1.0 (Xie et al., 2017a; Xie et al., 2017b; Kang, 2018). In addition, as discussed above, the TMRM-based mito-thermometry 2.0 is only fit for qualitative studies, because TMRM has a lighter molecule weight and smaller size (R) than Rh800 so that TMRM has a faster (∼23% experimentally estimated) transmembrane diffusion (D) obeying to the Stokes-Einstein relation (D∼1/R) under the depolarization. Moreover, another limitation of current mito-thermometry is not appropriate for *in vivo* study. RhB-ME and TMRM are unlikely to be evenly distributed in tissues or with depth *in vivo*, since both RhB-ME and TMRM are accumulated in mitochondrial matrix according to Nernst equation so that most of RhB-ME and TMRM would like to be absorbed by epithelial cells and hepatocytes when dyes were delivered via intravenous injection, or the vicinal cells of dye-injected tissue region, such as brain.

Interestingly, the results demonstrated that the heterogenous thermogenesis evoked by the β3 adrenoceptor agonist CL316, 243 only used overall up to ∼46% of the thermogenic capacity evoked by CCCP, which could explain the failure of clinical trials for obesity interventions as discussed in our previous reports (Xie et al., 2017b). On the other hand, the results demonstrated that the maximum thermogenesis evoked by NE + oligomycin A used up to ∼79% of the thermogenic capacity, which suggested the maximum thermogenic capacity under physiological conditions by inhibiting the proton-ATPase function of the mitochondrial complex V, such as under the cold activation of sympathetic nerve and the co-release of sympathetic transmitters (NE and ATP) (Xie et al., 2017b). One straightforward explanation for the conservation of thermogenic capacity is that it is extremely important to maintain MMP for mitochondrial function and avoid mitochondrial unfolded protein response, so that the MMP changes in brown adipocytes induced by NE + oligomycin A (Supplementary Data S4) or NE + ATP (Xie et al., 2017b) were mild.

## Data Availability

The raw data supporting the conclusions of this article will be made available by the authors, without undue reservation.

## Abbreviations

Rh800, rhodamine 800; RhB-ME, rhodamine B methyl ester; TMRM, tetra-methyl rhodamine methyl ester; MMP, mitochondrial membrane potential; NE, norepinephrine.
