# Supplementary material for: A sensitive mitochondrial thermometry 2.0 and the availability of thermogenic capacity of brown adipocyte
Source: Front Physiol. 2022 Aug 24;13:977431. doi: 10.3389/fphys.2022.977431 (PMC9449420; doi:10.3389/fphys.2022.977431)
Supplement: Supplementary file 1 [file DataSheet1.PDF]

Supplementary Material

Supplemental Data include four supplementary figures.

1 Supplementary Figures and legends.

1.1 Supplementary Figure 1| The spectra of Rhodamine dyes.

Supplement data 1| The excitation and emission spectra of Rhodamine dyes

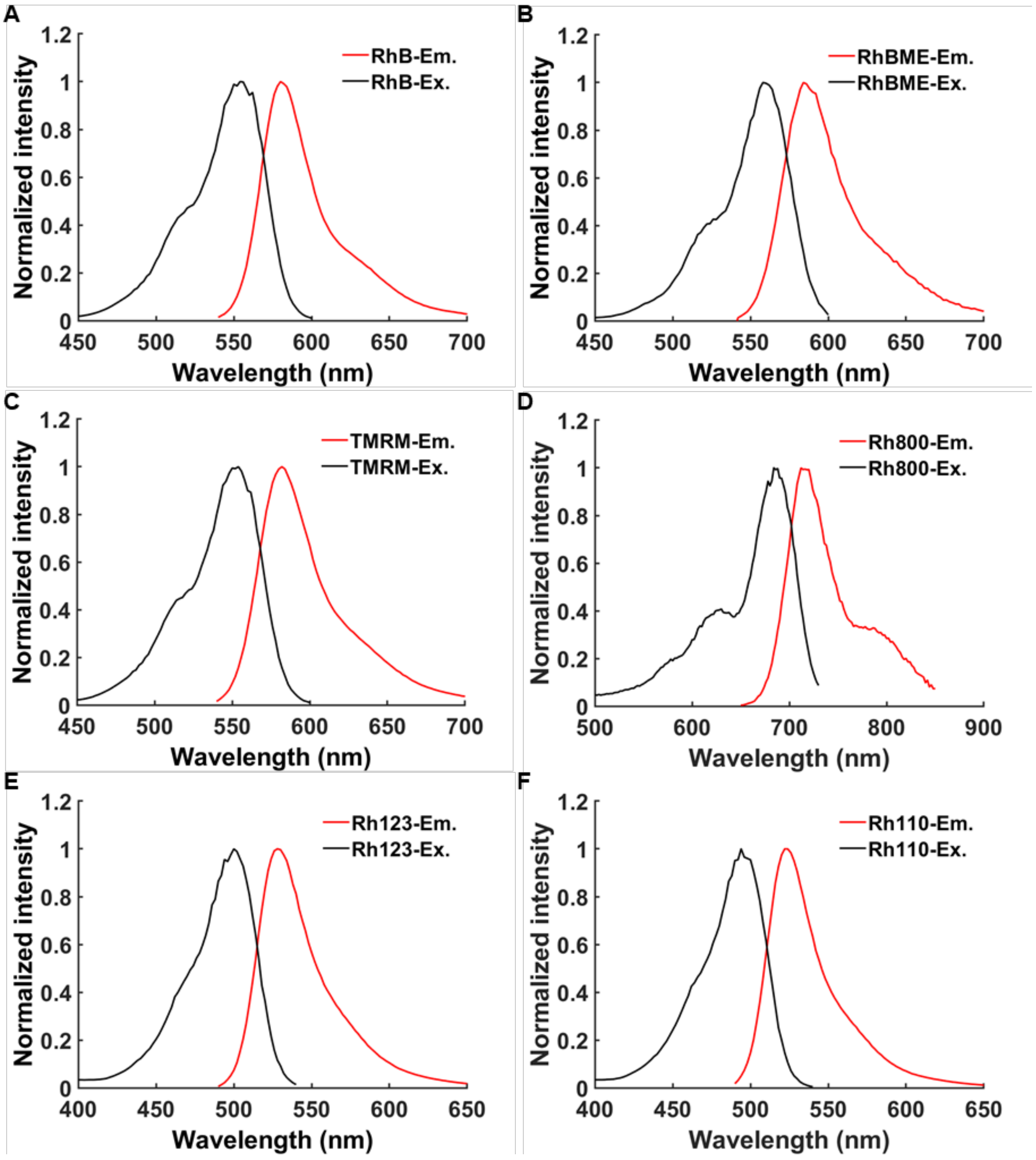

A-F, the excitation (black sold line) and emission (red sold line) spectra of RhB (**A**), RhB-ME (**B**), TMRM (**C**), Rh800 (**D**), Rh123 (**E**) and Rh110 (**F**). The Spectrum of RhB-ME (**B**) or TMRM (**C**) was well separated from that of Rh800 (**D**), but overlapped with that of Rh123 (**E**), therefore, Rh800 (**D**) rather than Rh123 (**E**) is used for the ratiometric mito-thermometries with RhB-ME (**B**) or TMRM (**C**). For spectral analysis, the concentrations of all dyes were used at 10 μM. For clarity and comparison, the spectra of RhB (**A**), RhB-ME (**B**), Rh800 (**D**) and Rh110 (**F**) were reused to be more informative from our previous work[9].

1.2 Supplementary Figure 2| The spectra of Rhodamine dyes in various pH.

Supplement data 2| The spectra of Rhodamine dyes in various pH

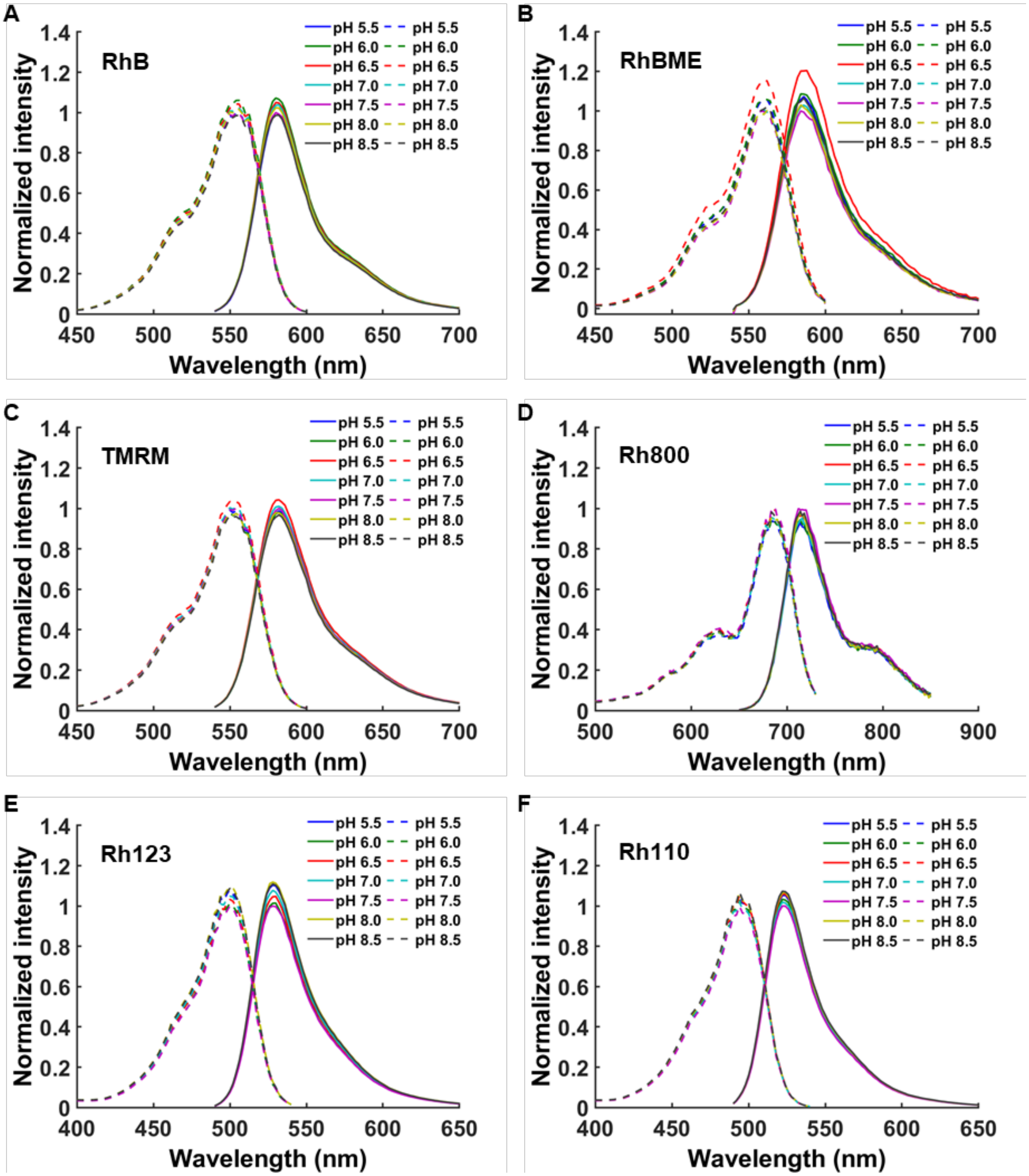

A-F, the excitation (dashed line) and emission (solid line) spectra of RhB (**A**), RhB-ME (**B**), TMRM (**C**), Rh800 (**D**), Rh123 (**E**) and Rh110 (**F**) in different pH from 5.5 to 8.5. These rhodamine dyes were relatively stable under various PH. For spectral analysis, the concentration of all dyes were used at 10 μM.

1.3 Supplementary Figure 3| The emission spectra of Rhodamine dyes at different temperatures.

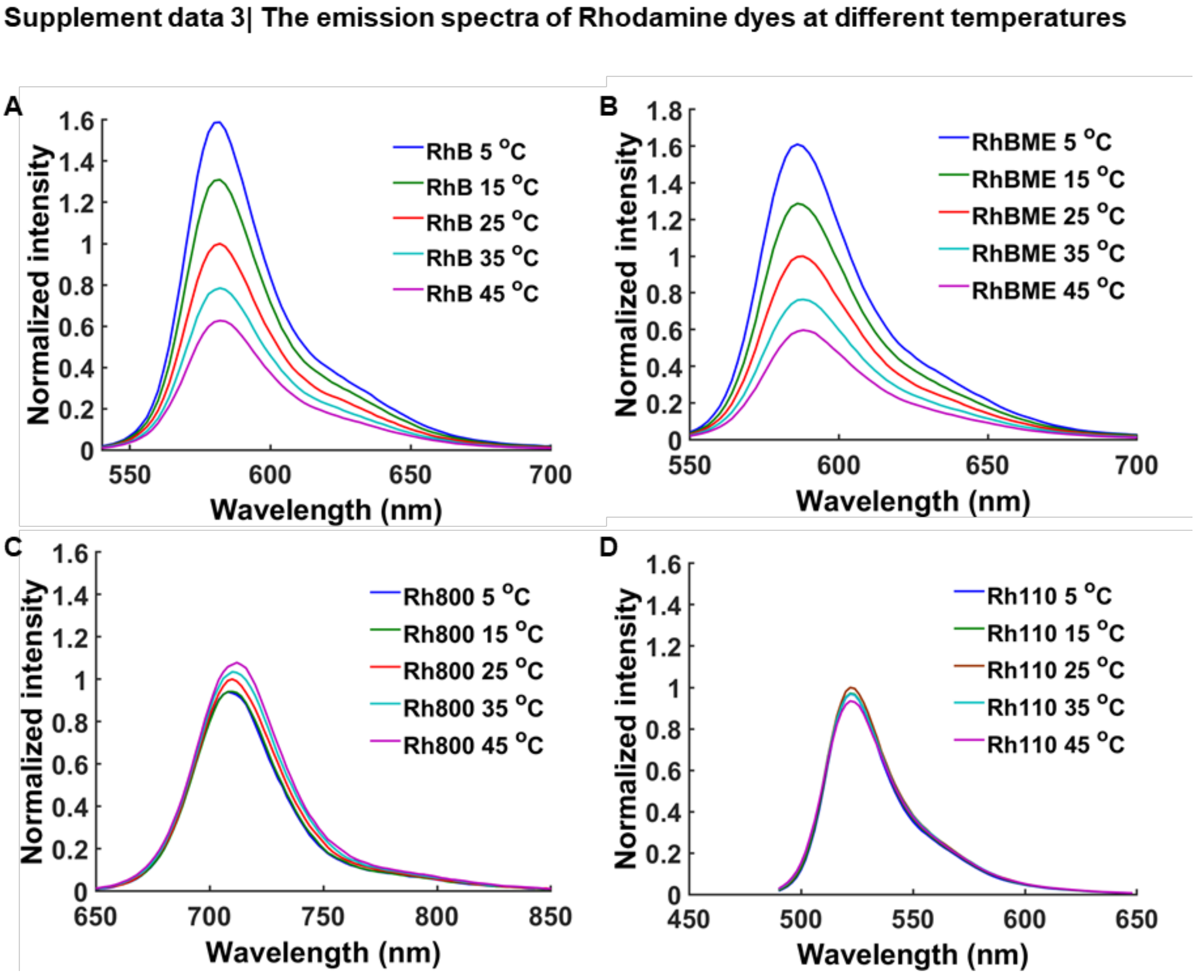

A-D, The emission spectra of of RhB (**A**), RhB-ME (**B**), Rh800 (**C**), Rh110 (**D**) from 5 °C to 45 °C, respectively. The fluorescence intensity of RhB (**A**) and RhB-ME (**B**) decreased as temperature increased, while the fluorescence intensity of Rh800 (**C**) and Rh110 (**D**) was not affected by temperature. For spectral analysis, the concentrations of all dyes were used at 10 μM. For clarity, the data were also collected and reprodued from our previous work[9].

1.4 Supplementary Figure 4| The heterogeneous responses in the brown adipocytes under NE stimulation

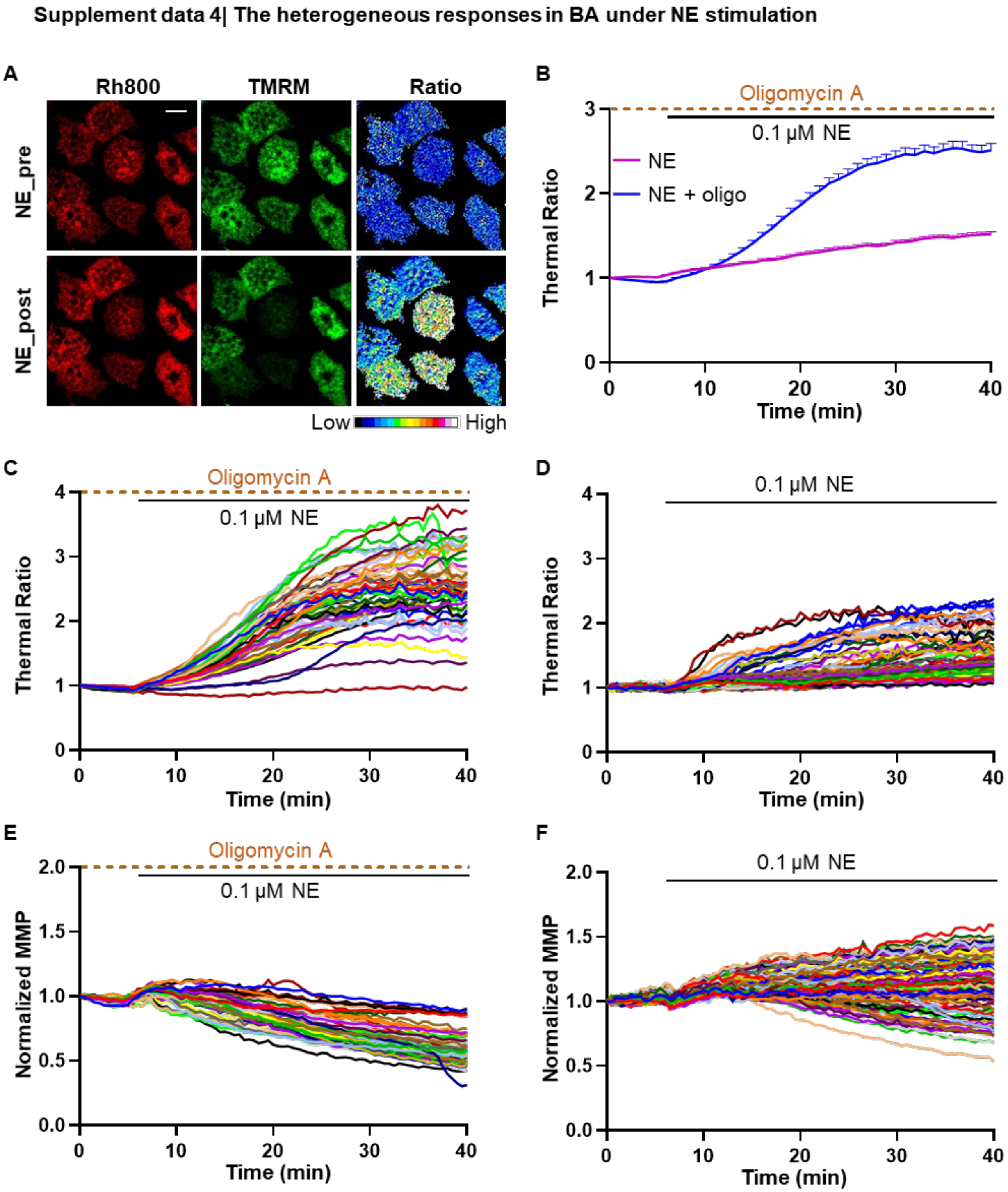

A, the representative fluorescence and thermal ratio images of brown adipocytes before and after the treatment with 0.1 µM of NE. It showed the heterogeneous thermogenic responses and MMP dynamics in brown adipocytes. Scale bar, 20 µm. **B**, the averaged thermogenic responses of the brown adipocytes treated by NE (purple line, n = 137) and NE + oligomycin A (blue line, n = 47). The result showed that oligomycin A increased the thermogenic efficacy of NE. All data points

represented mean + s.e.m. **C-D**, the thermogenic responses in brown adipocytes evoked by NE + oligomycin A (**C**) and NE (**D**), respectively. NE + oligomycin A evoked the thermogenic effect in almost all brown adipocytes, while NE only partially induced thermogenic responses. **E-F**, the raw data plots of MMP changes in brown adipocytes treated with NE + oligomycin A (**E**) and NE (**F**), respectively. The results demonstrated that NE + oligomycin A induced strong mitochondrial depolarization, while NE stimulation showed heterogenous MMP changes (depolarization and hyperpolarization). Each colored trace represented a single brown adipocyte in **C-F**.
